# A Good Way to Reduce Screening for Retinopathy of Prematurity: Development of the ROP Model in a China Preterm Population

**DOI:** 10.3389/fped.2021.697690

**Published:** 2021-06-30

**Authors:** Wenqian Ding, Chenghan Luo, Xinru Cheng, Zanyang Shi, Mengyuan Lei, Junbo Rong, Min Song, Wenjun Cao, Jingdi Zhang, Jian Ge, Mengmeng Wang, Yixia Zhang, Peige Xia, Li Wang, Yufeng Liu, Qian Zhang

**Affiliations:** ^1^Neonatal Intensive Care Unit, The First Affiliated Hospital of Zhengzhou University, Zhengzhou, China; ^2^Orthopeadics Department, The First Affiliated Hospital of Zhengzhou University, Zhengzhou, China; ^3^Health Care Department, The First Affiliated Hospital of Zhengzhou University, Zhengzhou, China; ^4^Ophthalmology Department, The First Affiliated Hospital of Zhengzhou University, Zhengzhou, China; ^5^Children Health Care Department, Children's Hospital Affiliated of Zhengzhou University, Zhengzhou, China; ^6^Pediatrics Department, The First Affiliated Hospital of Zhengzhou University, Zhengzhou, China

**Keywords:** retinopathy of prematurity, preterm, screening, China, NT-ProBNP

## Abstract

**Importance:** Retinopathy of prematurity (ROP) is a preventable cause of blindness in children. Without treatment, more than 45% of eyes may suffer permanent vision loss. Current ROP screening guidelines, which include a range of birth weights (BWs) and gestational ages (GAs), may require screening many low-risk preemies who might develop severe ROP.

**Method:** All high-risk infants in the neonatal intensive care unit (NICU) of the First Affiliated Hospital of Zhengzhou University from 2017 to 2021 were included in this retrospective cohort study. Each of the 27 candidate risk factors was evaluated in univariate analysis and adjusted for known risk factors (i.e., GA and BW). The significant results were analyzed in a backward selection multivariate logistic regression model. Receiver operating characteristic (ROC) curves and a nomogram were drawn.

**Results:** The study included 2,040 infants who underwent ROP screening. The weight gain rate [OR, 2.65; 95% confidence interval (CI), 1.49–1.21 ≤ 12 g/d vs. > 18 g/d; *P* = 0.001], blood transfusion (OR, 2.03; 95% CI, 1.14–3.64; *P* = 0.017), invasive mechanical ventilation (OR, 1.74; 95% CI, 1.15–2.66; *P* = 0.009) and N-terminal segment of pro-B-type natriuretic peptide (NT-proBNP) ≥ 25,000 ng/L (OR, 1.51; 95% CI, 1.00–2.28; *P* = 0.048) were four new statistically independent risk factors in addition to GA and BW. The area under the curve (AUC) of the final multivariate model was 0.90 (95% CI, 0.88–0.92; *P* < 0.001).

**Conclusions and Relevance:** These findings add to our understanding of ROP screening because they include all eligible infants rather than only high-risk infants, as in previous studies. Under the control of BW and GA, low weight gain rate, increased number of blood transfusion, invasive mechanical ventilation and NT-proBNP ≥ 25,000 ng/L were “new” statistically independent risk factors for ROP. The ROP risk can be calculated manually or represented by a nomogram for clinical use.

## Introduction

Retinopathy of prematurity (ROP) is a serious vascular proliferative disease of the retina in premature infants that can lead to visual impairment or blindness in children. This is mainly due to the mismatch between the supply and demand of oxygen in the retina (1, 2). ROP can usually be effectively treated if diagnosed within an urgent time window (3). The population of infants at risk for ROP varies by geographic region (3). In developed countries, the highest-risk infants are those with gestational age (GA) <28 weeks and birth weight (BW) <1,000 g (4, 5), while in areas where the quality of neonatal intensive and ophthalmological care varies, more mature infants with BW up to 2,000 g and GA up to 37 weeks may also develop severe ROP (6, 7). The increase in ROP in middle-income countries coincides with the increase in the survival of very low birth weight infants, known as the “third ROP epidemic” (8, 9).

The current guidelines for ROP screening in the United States recommend ROP screening for infants with BW <1,501 g or GA <30 weeks. Examinations are also recommended for slightly older (1,500 g ≤ BW <2,000 g) or more mature (>30-week GA) infants who are considered by the attending physician to be “clinically unstable” (10). In China, where ROP can also occur in large infants, screening is usually performed within 4–10 weeks after birth but it should be performed at 3 weeks after birth if BW >2,000 g (11). These standards have led to many low-yield screenings of larger, more mature births.

The fundoscopy used in ROP screening has proven to be painful (12, 13). ROP screening requires many infants to undergo a series of uncomfortable intensive diagnostic eye examinations, <10% of which need treatment (3). One way to reduce the number of ophthalmic examinations is to develop screening strategies to more accurately identify infants at high risk. Reducing exposure to pain is the goal of neonatal care, especially for premature infants. In addition, it is important to reduce the workload and financial costs of wards. As screening and treatment face different challenges, there is an urgent need to improve global ROP management strategies.

Some people consider ROP to be a multifactorial disease (14–16). As a result, there are many candidate risk factors, such as weight growth rate, multiple births, sex, mechanical ventilation, respiratory distress syndrome (RDS), blood transfusion, intraventricular hemorrhage, and maternal risk factors (17–21). These studies only conducted univariate analysis and did not adjust for confounding variables (17–19).

The N-terminal segment of pro-B-type natural peptide (NT-proBNP) is an inactive substance secreted by cardiac cells when the cardiac volume or pressure load changes. NT-proBNP plays an important role in the diagnosis and treatment of adult heart failure and other diseases. It has been reported that high level of urinary NT-proBNP is associated with severe ROP (22, 23). High levels of serum NT-proBNP may lead to retinal injury, suggesting that NT-proBNP may be a marker of small vessel disease (24).

The objectives of this study were to screen and remove negligible potential risk factors for ROP, identify statistically independent risk factors and develop a nomogram for clinical use. Through these findings, we can build better risk-based screening models.

## Materials and Methods

This retrospective cohort study included preterm infants born at the First Affiliated Hospital of Zhengzhou University between January 2017 and February 2021. Through the hospital medical record system, data on the admission, diagnosis and treatment of infants, as well as data on examinations and surgeries performed during hospitalization were available.

### Ethics Statement

This study was approved by the Ethics Department of the First Affiliated Hospital of Zhengzhou University. Informed consent for ophthalmic examination was obtained from the eligible infants' parents/guardians.

### Identification of the Cohort of Premature Infants

From January 2017 to February 2021, a total of 2040 high-risk infants in the neonatal intensive care unit (NICU) were included in the study according to national guidelines (25). “High risk infants” is defined as follows: 1. BW <2,000 g; 2. GA ≤ 34 weeks; 3. selected premature infants with BW ≥2,000 g or GA >34 weeks, suffering from diseases requiring cardiopulmonary support, long-term oxygen therapy and respiratory suspension, anemia requiring blood transfusion and newborn septicemia, or considered to be at high risk by their attending pediatrician. Enrolled infants survived at least until discharge after the initial hospital stay and retinal examinations reached the postmenstrual age (PMA) of 45 weeks.

We analyzed the characteristics of infants with ROP diagnosed at the end of the study or before discharge. The stages of retinopathy are classified according to the International Classification of Retinopathy of Prematurity. The weight gain rate was calculated by dividing the weight gain from day 7 to the first screening by the number of days of life from day 7 to the first screening. Weight in the first week of life was excluded from the analysis because very low-BW infants typically lose weight during this time.

Our research center attaches great importance to the management of NT-proBNP. The children included in the study were monitored for NT-proBNP at least once after birth, and their NT-proBNP levels were regularly reviewed if necessary. If the level of NT-proBNP at a certain time was obviously abnormal and inconsistent with the clinical situation, we conducted a second test at that time. The measurement range of NT-proBNP is 5~35,000 ng/L. The grade of NT-proBNP was determined based on the highest value measured during the infant's hospital stay.

Patients with BW below the 10th percentile were classified as small for gestational age (SGA) (26). Invasive mechanical ventilation refers to the establishment of an invasive artificial airway through endotracheal intubation or tracheotomy for mechanical ventilation. Blood transfusion is defined as a single transfusion or multiple transfusion therapy during hospitalization, including red blood cell suspension transfusion, platelet transfusion, plasma transfusion, etc. We defined bronchopulmonary dysplasia (BPD) as the oxygen demand at 36 weeks GA (27). Prenatal steroid use meant taking any steroid drug before delivery. Other relevant clinical details, such as sex, maternal risk factors (including maternal gestational diseases) and complications of preterm birth, were recorded and collected.

### Data Analyses

For the sample size, when using logistic regression, the number of events should be at least 10 times the number of significant independent variables. The total number of observed samples should be at least 20–30 times the number of independent variables. We first used univariate analysis to analyse the predictive factors of ROP. Then, 27 candidate risk factors for ROP were analyzed one at a time in a logistic regression model while adjusting for known risk factors: GA and BW. Candidate risk factors associated with a significantly increased risk were included in the multiple logistic regression model, which also included the known risk factors listed above. Finally, backward stepwise selection was performed to identify independent risk factors. *P*-values < 0.05 were considered statistically significant. The odds ratio (OR) and 95% confidence interval (CI) associated with each predictive variable were calculated from the logistic regression model. The AUC was used to evaluate the model's prediction effectiveness. We also calculated the sensitivity and specificity corresponding to each cut point of the ROP prediction probability predicted by the final multiple model. Single-factor analysis and a multifactor regression model were carried out in SPSS 26.0. The final logistic model was transformed into a nomogram by using mathematical software (R 3.63).

## Results

In this study, the mean GA was 32 weeks (SD, 2; range, 25–37). The mean BW was 1,855 g (SD, 575; range, 600–4,800). Of the 2,040 infants included in the study, 768 (37.6%) received at least two ophthalmic examinations. The median PMA for the first ophthalmic examination was 35.0 weeks. The ophthalmic examination to confirm ROP was performed with a median PMA of 35.2 weeks. A total of 241 (11.8%) infants developed ROP and approximately 46 (2.3%) children needed treatment for ROP. Both rates are consistent with previous studies (3, 28).

Table 1 shows the known and “new” risk factors throughout the study period. The demographic predictors associated with ROP were a low BW (*P* < 0.001) and a low GA (*P* < 0.001) but not sex (*P* = 0.927). Invasive mechanical ventilation (*P* < 0.001), blood transfusion (*P* < 0.001) and low weight gain rate (*P* < 0.001) were associated with ROP. NT-proBNP was divided into four levels: ≥25,000 ng/L, ≥20,000 ng/L, ≥15,000 ng/L, and ≥10,000 ng/L. The four levels were statistically significant (*P* < 0.001).

**Table 1:** Characteristics of the 2,040 premature infants included in the study.

**Characteristic (*N* = 2,040)**	**No. of infants at risk**	**Incidence rate of ROP (Yes vs. No)^**a**^**	***P*-value**
**Gestational age, wk**^**b**^			
≤ 28	70 (3.4)	75.7%	<0.001
28–30	224 (11.0)	43.3%	
30–32	380 (18.6)	15.3%	
>32	1,366 (67.0)	2.4%	
**Birth weight, g**^**c**^			
<1,000	74 (3.6)	71.6%	<0.001
1,000–1,500	527 (25.8)	27.9%	
≥1,500	1,439 (70.5)	2.8%	
NT-proBNP ≥ 25,000 ng/L	293 (14.4)	34.6 vs. 7.5%	<0.001
NT-proBNP ≥ 20,000 ng/L	342 (16.8)	32.5 vs. 7.7%	<0.001
NT-proBNP ≥ 15,000 ng/L	434 (21.3)	28.8 vs. 7.2%	<0.001
NT-proBNP ≥ 10,000 ng/L	601 (29.5)	25.0 vs. 6.3%	<0.001
Male	1,123 (55.0)	11.8 vs. 11.9%	0.927
Intrauterine asphyxia	28 (1.4)	28.6 vs. 11.6%	0.013
FGR	148 (7.3)	20.3 vs. 11.2%	0.001
PROM	492 (24.1)	13.6 vs. 11.2%	0.155
Cesarean section	1,740 (85.3)	10.2 vs. 21.0%	<0.001
Multiple births	515 (25.2)	11.1 vs. 12.1%	0.544
Placenta previa	440 (21.6)	11.1 vs. 12.0%	0.619
Nuchal cord	493 (24.2)	11.8 vs. 11.8%	0.969
Amniotic fluid pollution	349 (17.1)	18. vs. 10.3%	<0.001
**Apgar 1 min, score**			
1–3	49 (2.4)	30.6%	<0.001
4–7	381 (18.7)	24.9%	
8–10	1,610 (78.9)	8.1%	
**Apgar 5 min, score**			
1–3	10 (0.5)	20.0%	<0.001
4–7	111 (5.4)	38.7%	
8–10	1,919 (94.1)	10.2%	
Gestational hypertension	698 (34.2)	13.6 vs. 10.9%	0.070
GDM	371 (18.2)	11.9 vs. 11.8%	0.976
Embryo transfer	363 (17.8)	14.0 vs. 11.3%	0.145
Bad pregnancy or reproduction history	283 (13.9)	16.3 vs. 11.1%	0.013
Prenatal steroid	568 (27.8)	13.2 vs. 11.3%	0.227
Pulmonary surfactant therapy	668 (32.7)	28.1 vs. 3.9%	<0.001
Invasive mechanical ventilation	240 (11.8)	45.8 vs. 7.3%	<0.001
Non-invasive mechanical ventilation	1,036 (50.8)	11.2 vs. 12.5%	0.381
Nasal catheter oxygen inhalation	35 (1.7)	0.0 vs. 12.0%	0.055
Blood transfusion	1,018 (49.9)	21.7 vs. 2.0%	<0.001
**Weight gain rate, g/d**			
≤ 12	138 (6.8)	29.0%	<0.001
12–18	190 (9.3)	31.6%	
>18	1,712 (83.9)	8.2%	
Asphyxia	369 (18.1)	28.2 vs. 8.2%	<0.001
Apnea	92 (4.5)	31.5 vs. 10.9%	<0.001
RDS	1,044 (51.2)	20.6 vs. 2.6%	<0.001
Cerebral hemorrhage	1,256 (61.6)	14.1 vs. 8.2%	<0.001
Coagulation dysfunction	1,060 (52.0)	16.4 vs. 6.8%	<0.001
BPD	229 (11.2)	27.1 vs. 7.1%	<0.001
SGA	420 (20.6)	16.7 vs. 10.6%	0.001

*GDM, gestational diabetes mellitus; FGR, fetal growth restriction; PROM, premature rupture of membranes; SGA, small for gestational age; BPD, bronchopulmonary dysplasia; RDS, respiratory distress syndrome*.

a *Incidence rate of ROP among infants when the risk factor is present (yes) vs. not present (no)*.

b *Median, Mean (SD; range), 33,33 (2; 25–37)*.

c *Median, Mean (SD; range), 1,800, 1,855 (575; 600–4,800)*.

Cesarean section (*P* < 0.001) and surfactant therapy (*P* < 0.001) were associated with ROP but not multiple births (*P* = 0.544). Maternal gestational diseases including gestational hypertension (*P* = 0.070) and gestational diabetes mellitus (GDM) (*P* = 0.970), were not associated with the risk of ROP. Intrauterine asphyxia (*P* = 0.013), fetal growth restriction (*P* = 0.001), and amniotic fluid contamination (*P* < 0.001) were associated with ROP. Neonatal diseases associated with a high risk of ROP included cerebral hemorrhage (*P* < 0.001), asphyxia (*P* < 0.001), apnea (*P* < 0.001), respiratory distress syndrome (RDS) (*P* < 0.001), BPD (*P* < 0.001) and coagulation dysfunction (*P* < 0.001).

Table 2 presents the results of various logistic regression analyses to evaluate the potential “new” risk factors for ROP. The variables in Table 1 with *P* < 0.05 were selected. Various potential risk factors were independently analyzed, after adjustment for known ROP risk factors (i.e., GA and BW). Crude OR, adjusted OR and 95% CI were reported. The analysis results (Table 2) were ranked according to the adjusted statistical significance; therefore, the seven statistically significant results (*P* < 0.05) are regarded as the first seven items.

**Table 2:** Univariate Logistic Regression Results of the Significant Risk Factors Reported in Table 1.

**Specification**	**Crude OR**		**Adjusted OR**^**a**^	
	**OR (95% CI)**	***P*-value**	**OR (95% CI)**	***P*-value**
**Weight gain rate, g/d**				
≤ 12	4.55 (3.03–6.83)	<0.001	3.68 (2.11–6.40)	<0.001
12–18	5.14 (3.62–7.31)	<0.001	2.32 (1.48–3.64)	<0.001
>18	1[Reference]	<0.001		<0.001
FGR	2.03 (1.32–3.10)	0.001	2.52 (1.42–4.47)	0.002
Invasive mechanical ventilation	10.78 (7.91–14.70)	<0.001	2.35 (1.60–3.44)	<0.001
NT-proBNP ≥ 25,000 ng/L	7.42 (5.52–9.97)	<0.001	2.18 (1.52–3.13)	<0.001
SGA	1.69 (1.25–2.29)	0.001	1.80 (1.11–2.92)	0.017
Blood transfusion	13.89 (8.71–22.16)	<0.001	1.79 (1.03–3.11)	0.039
BPD	12.48 (9.11–17.10)	<0.001	1.67 (1.13–2.47)	0.010
NT-proBNP ≥ 20,000 ng/L	5.80 (4.34–7.73)	<0.001	1.59 (1.12–2.28)	0.010
NT-proBNP ≥ 15,000 ng/L	5.20 (3.92–6.88)	<0.001	1.49 (1.05–2.10)	0.026
NT-proBNP ≥ 10,000 ng/L	4.93 (3.72–6.53)	<0.001	1.47 (1.04–2.07)	0.030
**Apgar 5 min, score**				
1–3	2.20 (0.46–10.42)	0.321	1.75 (0.29–10.70)	0.546
4–7	5.56 (3.69–8.37)	<0.001	1.36 (0.81–2.28)	0.245
8–10	1[Reference]	<0.001		0.433
Bad pregnancy or reproduction history	1.55 (1.10–2.20)	0.013	1.56 (0.99–2.45)	0.055
Asphyxia	4.39 (3.30–5.85)	<0.001	1.39 (0.97–2.00)	0.075
Apnea	3.77 (2.37–5.99)	<0.001	1.38 (0.79–2.41)	0.263
Coagulation dysfunction	2.68 (1.99–3.60)	<0.001	1.34 (0.94–1.91)	0.103
**Apgar 1 min, score**				
1–3	4.98 (2.64–9.38)	<0.001	1.32 (0.61–2.84)	0.477
4–7	3.75 (2.80–5.03)	<0.001	1.17 (0.81–1.70)	0.398
8–10	1[Reference]	<0.001		0.597
Amniotic fluid pollution	2.02 (1.48–2.76)	<0.001	1.29 (0.87–1.89)	0.201
Pulmonary surfactant therapy	9.75 (7.06–13.45)	<0.001	1.21 (0.79–1.84)	0.385
RDS	9.68 (6.37–14.69)	<0.001	1.11 (0.66–1.86)	0.692
Cesarean section	0.43 (0.31–0.59)	<0.001	1.04 (0.68–1.61)	0.844
Cerebral hemorrhage	1.85 (1.37–2.49)	<0.001	1.02 (0.71–1.46)	0.926
Intrauterine asphyxia	3.05 (1.33–7.01)	0.008	0.68 (0.26–1.74)	0.417

*CI, confidence interval; OR, odds ratio; FGR, fetal growth restriction; SGA, small for gestational age; BPD, bronchopulmonary dysplasia; RDS, respiratory distress syndrome*.

a *All analyses only adjusted for known risk factors: GA, SGA. The analysis results are ranked according to the adjusted statistical significance*.

Table 3 shows the results of multivariate logistic regression analysis to identify independent predictors of potential risk factors for ROP. The reported model is the result of backward selection, removing one insignificant risk factor at a time. All known and new significant risk factors were examined. Demographic and clinical factors independently associated with ROP include: low GA (OR, 13.11; 95% CI, 5.90–29.15 ≤ 28 vs. > 32 weeks; *P* < 0.001), low BW (OR, 9.65; 95% CI, 4.64–20.06 <1,000 vs. ≥ 1,500 g; *P* < 0.001), low weight growth rate (OR, 2.65; 95% Cl, 1.49–4.72 ≤ 12 vs. > 18 g/d; *P* = 0.001), blood transfusion (OR, 2.03; 95% CI, 1.14–3.64; *P* = 0.017), invasive mechanical ventilation (OR, 1.74; 95% CI, 1.15–2.66; *P* = 0.009) and NT-proBNP ≥25,000 ng/L (OR, 1.51; 95% Cl, 1.00–2.28; *P* = 0.048). Low GA was the most important risk factor.

**Table 3:** Multiple Logistic Regression Estimates Based on Backward Selection Eliminating from a Model with Known Risk Factors (Gestational Age, Birth Weight,) and Significant Risk Factors Reported in Table 2^a^.

**Specification**	**β**	**OR (95% CI)**	***P*-value**
**Gestational age, wk**			
≤ 28	2.57	13.11 (5.90–29.15)	<0.001
28–30	1.72	5.61 (3.12–10.09)	<0.001
30–32	1.03	2.80 (1.66–4.74)	<0.001
>32		1	[Reference]
**Birth weight, g**			
<1,000	2.27	9.65 (4.64–20.06)	<0.001
1,000–1,500	0.98	2.67 (1.640–4.34)	<0.001
≥1,500		1	[Reference]
**Weight gain rate, g/d**			
≤ 12	0.98	2.65 (1.49–4.72)	0.001
12–18	0.66	1.94 (1.21–3.11)	0.006
>18		1	[Reference]
Blood transfusion	0.71	2.03 (1.14–3.64)	0.017
Invasive mechanical ventilation	0.56	1.74 (1.15–2.66)	0.009
NT-proBNP ≥ 25,000 ng/L	0.41	1.51 (1.00–2.28)^b^	0.048
Constant	−4.40	0.01	<0.001

*CI, confidence interval; OR, odds ratio; PMA, postmenstrual age; ROP, retinopathy of prematurity*.

a *The multivariate model started with all the significant predictors in Tables 1, 2. The model went through the stepwise selection by keeping only the statistical significant variables in the final multivariate model*.

b *If three decimal places are reserved, the 95% CI is 1.004–2.282*.

**Figure 1:**
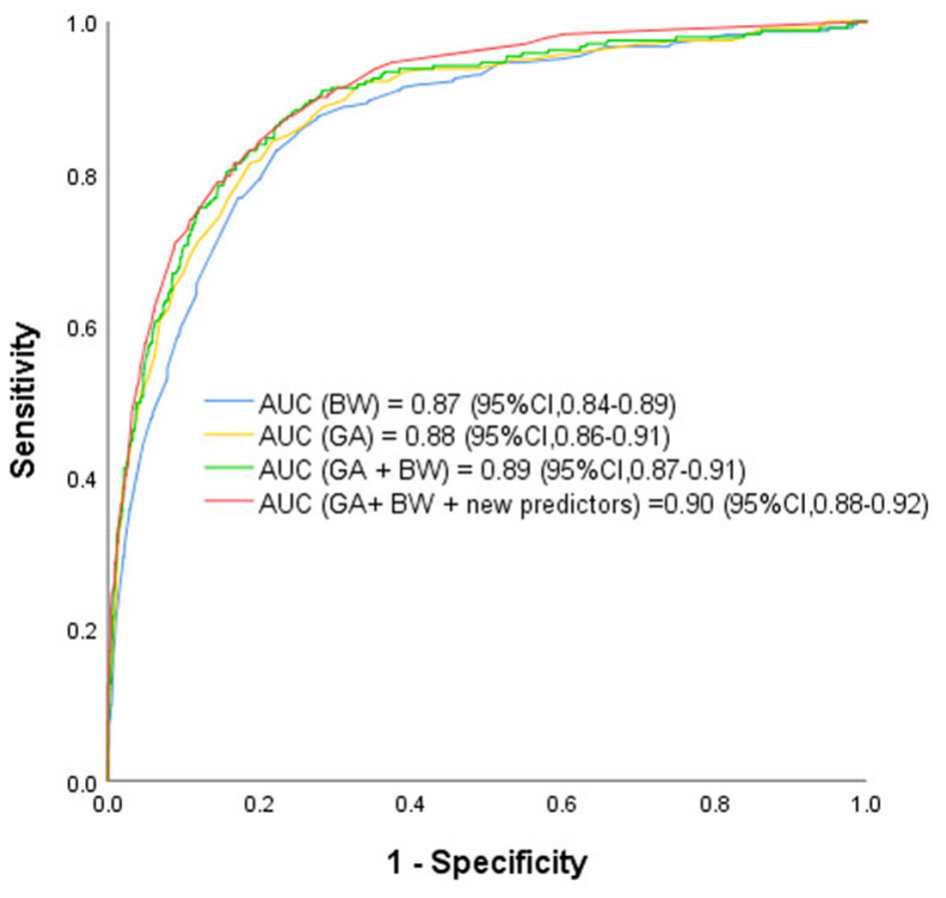
Receiver Operating Characteristic Curves rom the Prediction Models Using Various Combinations of Predictors for Retinopathy of Prematurity Based on the Final Prediction Model. GA, gestational age; BW, birth weight; BT, blood transfusion; IMV, invasive mechanical ventilation; WGR, weight gain rate. The receiver operating characteristic curves from retinopathy of prematurity prediction models using various combinations of predictors including known predictors (birth weight, gestational age, and new predictors (weight gain rate, blood transfusion, invasive mechanical ventilation and NT-proBNP ≥ 25,000 ng/L). AUC indicates area under receiver operating characteristic curve.

**Figure 2:**
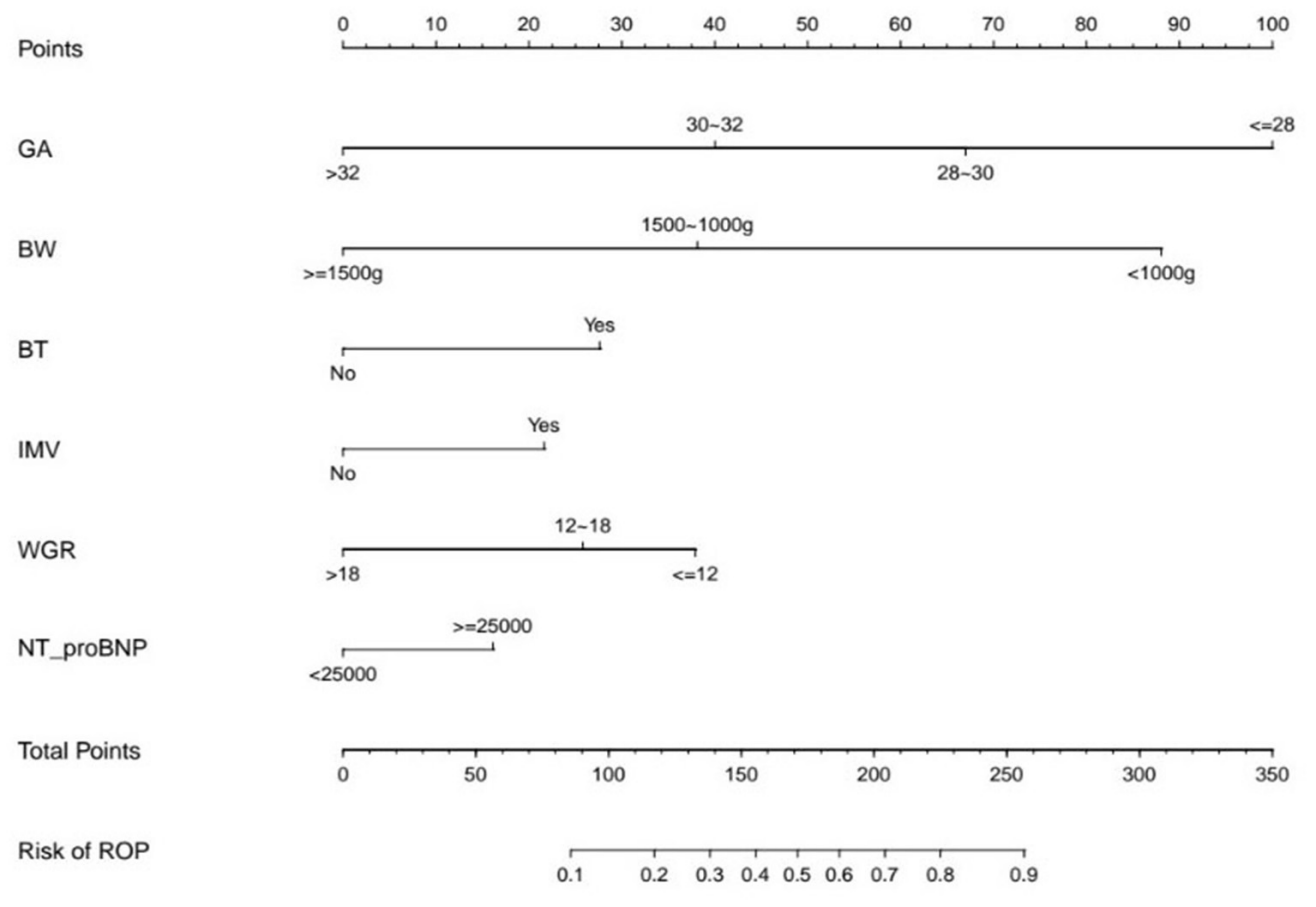
The Nomogram Based on the Final Prediction Model. GA, gestational age; BW, birth weight; BT, blood transfusion; IMV, invasive mechanical ventilation; WGR, weight gain rate; ROP, retinopathy of prematurity.

ROCs were derived from multiple models with and without newly identified risk factors, as shown in Figure 1. The AUCs of BW and GA for predicting ROP were 0.87 (95% CI, 0.84–0.89) and 0.88 (95% CI, 0.86–0.91), respectively. The AUC of combining BW with GA was 0.89 (95% CI, 0.87–0.91). When the new risk factors were added to the final multivariate model, the AUC was 0.90 (95% CI, 0.88–0.92), which was significantly better than the prediction by demographic characteristics. Table 4 shows the sensitivity and specificity of different cut-points based on ROP prediction probability. A nomogram was created based on the regression equation for the base model (Figure 2).

**Table 4:** Sensitivity and specificity for chosen cut points based on predicted risk from the multiple model with both known and new risk factors included.

**Cut point**	**Sensitivity (*N* = 248)**	**Specificity (*N* = 1,792)**
≥0.02	244 (98.3%)	717 (40.0%)
≥0.05	224 (90.5%)	1,272 (71.0%)
≥0.10	202 (81.3%)	1,486 (82.9%)
≥0.15	195 (78.8%)	1,518 (84.7%)
≥0.20	181 (73.0%)	1,600 (89.3%)
≥0.50	100 (40.2%)	1,749 (97.6%)

*ROP, retinopathy of prematurity*.

## Discussion

It has been proven that ROP is a multifactorial disease (17–21). At present, many ROP prediction models and algorithms have been developed and have demonstrated the potential to reduce the number of ophthalmoscopies (for example, RW-ROP, severe ROP, TR-ROP) (29–33). In this study, we found that a model based on BW, GA, weight growth rate, blood transfusion, invasive mechanical ventilation, and NT-proBNP ≥ 25,000 ng/L could predict ROP well (AUC = 0.90).

This retrospective cohort study provides an informative overview of the incidence of ROP in high-risk premature infants, including infants who meet the current recommended screening criterial, as well as infants of large weight or gestational age who were screened due to the concerns of the neonatologist. We found that most ROP cases and almost all severe ROP cases occurred in immature, low BW infants. Of 1,439 infants with BW ≥ 1,500 g, only 41 (2.8%) had ROP. Of the 1,366 infants with GA >32 weeks, only 33 (2.4%) developed ROP.

Because this study covers a wide range of BW and GA, the population at risk for ROP could be more clearly identified in infants meeting current screening criteria compared with previous clinical trials and research data specifically focusing only on high-risk infants. The aim was to more accurately identify a small number of infants with ROP in the high BW or GA categories by using other demographic or higher risk indicators. If validated by independent studies, our predictive model can be used to stratify infants at high risk for ROP and reduce or eliminate testing for infants at very low ROP risk.

Low BW and GA are the main predictors of ROP (34–37). This study confirmed their independent correlation with ROP. As other studies have shown (34, 35), GA is more strongly correlated with ROP than BW (AUC of GA = 0.88, AUC of BW = 0.87).

B-type natriuretic peptide (BNP) can be used to assess cardiac insufficiency and to guide treatment. Biologically inactive NT-proBNP and BNP are produced and released in equal molar ratios, but the half-life is longer than that of BNP. Czernik et al. first reported that urinary NT-proBNP was associated with ROP, and the AUCs of the ratio of urinary NT-proBNP concentration to creatinine (UNBCR) on the 14 and 28th days after birth for predicting severe ROP were 0.938 (*P* = 0.027) and 0.954 (*P* = 0.021), respectively (23). Our previous study found that the level of NT-proBNP at different time points after birth in the ROP group was higher than that in the non-ROP group, and the serum level of NT-proBNP on the 14th day was significantly correlated with the occurrence of ROP (*P* < 0.001) (38). ROP is more common in infants with increased myocardial pressure or volume load, such as sepsis, blood transfusion, and patent ductus arteriosus (33, 39, 40). These conditions lead to BNP release and are associated with several groups of patients with elevated NT-proBNP (41, 42).

The potential physiological role of BNP itself in the development of ROP remains unclear. Vascular endothelial growth factor (VEGF) plays a key role in promoting vascular growth and remodeling in ROP. BNP and related type A natriuretic peptides (ANPs) appear to inhibit the activation of several key signaling molecules during VEGF-induced angiogenesis (43). ANP is also an effective inhibitor of vascular leakage and angiogenesis caused by VEGF (44). Retinal vessels have BNP receptors. Hypoxia can stimulate retinal epithelial cells to secrete BNP. High concentrations of NT-proBNP may be involved in retinopathy (22, 45, 46). Our results may provide a new research direction for predicting ROP in the future.

Recent studies have shown that using predictive models that include postnatal weight gain can significantly reduce the number of babies that need to be examined while also accurately identifying ROP infants (47). Low serum insulin-like growth factor-1 (IGF-1) is associated with slow weight gain. Low serum IGF-1 insufficiently activates retinal VEGF and leads to poor retinal vascular growth in the early postnatal period (48, 49). Previous studies on weight gain rates have focused on infants with GA ≤ 32 weeks. More mature infants can also experience poor weight gain due to various diseases. Therefore, this study included infants with a higher GA and BW, which can more comprehensively understand the impact of the weight growth rate on ROP.

Previous studies have found that blood transfusion is an independent risk factor for ROP. The main reasons for this include the following: (1) blood transfusion may increase IGF-1 levels, thereby stimulating retinal neovascularization; and (2) repeated infusion of adult-type hemoglobin with a low oxygen affinity leads to oxidative vascular damage that induces the development of ROP (18–53). At present, there are no national guidelines for neonatal blood transfusion in China. The decisions about neonatal transfusion treatment in the Chinese clinical environment are mainly based on experience or by referring to foreign transfusion guidelines. Studies have found that hospital size, the number of neonatal beds, areas and other factors are related to the blood transfusion rate of premature infants (54). As this study center is a critical neonatal treatment center in Henan Province of China, with 107 neonatal beds, it is understandable that the blood transfusion rate of premature infants in this study was 49.9% (Table 1).

How mechanical ventilation leads to ROP is controversial. Some studies have speculated that mechanical ventilation is only a confounding variable of oxygen supplementation (16, 20, 21, 53). In China (25), the indications for oxygen therapy for premature infants are clinical signs of respiratory distress, arterial oxygen partial pressure <50 mmHg or percutaneous oxygen saturation <85% during air inhalation. Our research center strictly restricts the use of mechanical ventilation. Our results showed that nasal catheter oxygen inhalation and noninvasive mechanical ventilation were not associated with ROP, whereas invasive mechanical ventilation was associated with ROP. This suggests that hyperbaric oxygen exposure and fluctuations of oxygen level during mechanical ventilation may be a real problem (16, 20).

This prediction model can effectively predict the possibility of ROP in infants. Including all of the predictors, the AUC was 0.90 (95% CI, 0.88–0.92), which is very good. When the probability of predicting ROP used 0.02 as the cut-off point, the sensitivity of the model was 98.3% and the specificity was 40.0%, which means that a large number of eye examinations (~35.3%) could be avoided, while most ROPs could still be detected.

### Strengths and Limitations

Advantages of this study include standard ROP examinations performed by study-certified ophthalmologists; and a comprehensive assessment of predictors, including demographic and clinical characteristics. However, the study is limited. Clinical and ophthalmic data were collected retrospectively. The timing of the subsequent funduscopies was based on the clinical decisions of different attending ophthalmologists and may affect the timing of the diagnosis of the stage of ROP. However, the doctors who perform fundus examinations have received professional training and use standardized international classifications for ROP staging. Therefore, we do not believe that the use of retrospective data would introduce substantial bias into this analysis. This study also excluded those who did not undergo fundus examination due to death or family financial factors. These exclusions may diminish the generality of our findings. In previous studies, some risk factors were continuous variables. However, our sample size was acceptable, and we were able to assess some risk factors, even for categorical variables. Finally, for similar reasons, the findings do not necessarily generalize to other parts of the world, where differences in neonatal care affect the ROP risk (7, 55).

## Conclusion

We identified predictors of ROP among the candidate risk factors, including BW, GA, weight gain rate, blood transfusion, invasive mechanical ventilation, and NT-proBNP. Serological markers for predicting the ROP risk are unclear. More studies are needed to verify the relationship between serum NT-proBNP and ROP, which will help to establish a more advanced risk-based ROP screening model. In addition, the low-risk characteristics of infants with a higher BW and GA who undergo ROP screening support making additional efforts to improve the specificity of risk assessment for these infants and ultimately to consider reassessing the current criteria. Additional risk factors not included in the statistical model may also contribute to the development of the ROP. Future research into ROP risk factors could evaluate other untested factors. The detection of type-1 ROP (treatable ROP) in a small group of infants with a higher BW and GA in the Chinese population will also be a focus of future research.

## Data Availability Statement

The original contributions presented in the study are included in the article/supplementary material, further inquiries can be directed to the corresponding authors.

## Ethics Statement

The studies involving human participants were reviewed and approved by the Ethics Department of the First Affiliated Hospital of Zhengzhou University. Written informed consent to participate in this study was provided by the participants' legal guardian/next of kin.

## Author Contributions

QZ and YL had full access to all of the data in the study and takes responsibility for the integrity of the data and the accuracy of the data analysis. WD and CL drafted the initial manuscript. WD, MS, WC, JZ, JG, and MW organized the database. All authors contributed to the article and approved the submitted version.

## Conflict of Interest

The authors declare that the research was conducted in the absence of any commercial or financial relationships that could be construed as a potential conflict of interest.
